# Supplementary material for: Designing Covalent Organic Framework‐Based Light‐Driven Microswimmers toward Therapeutic Applications
Source: Adv Mater. 2023 Apr 28;35(25):2301126. doi: 10.1002/adma.202301126 (PMC11475396; doi:10.1002/adma.202301126)
Supplement: Supplementary file 1 — Supporting Information [file ADMA-35-2301126-s003.pdf]

ADVANCED MATERIALS

Supporting Information

for *Adv. Mater.*, DOI 10.1002/adma.202301126

Designing Covalent Organic Framework-Based Light-Driven Microswimmers toward Therapeutic Applications

Varun Sridhar, Erdost Yildiz, Andrés Rodríguez-Camargo, Xianglong Lyu, Liang Yao, Paul Wrede, Amirreza Aghakhani, Birgul M. Akolpoglu, Filip Podjaski, Bettina V. Lotsch* and Metin Sitti**

Supporting Information

for

Designing Covalent Organic Framework-based Light-driven Microswimmers towards Therapeutic Applications

Varun Sridhar, Erdost Yildiz, Andrés Rodríguez-Camargo, Xianglong Lyu, Liang Yao, Paul Wrede, Amirreza Aghakhani, Birgul M. Akolpoglu, Filip Podjaski, Bettina V. Lotsch*, Metin Sitti**

This file includes:

Figures S1 to S5

Tables S1

Other Supplementary Materials for this manuscript include the following:

Videos S1 to S6

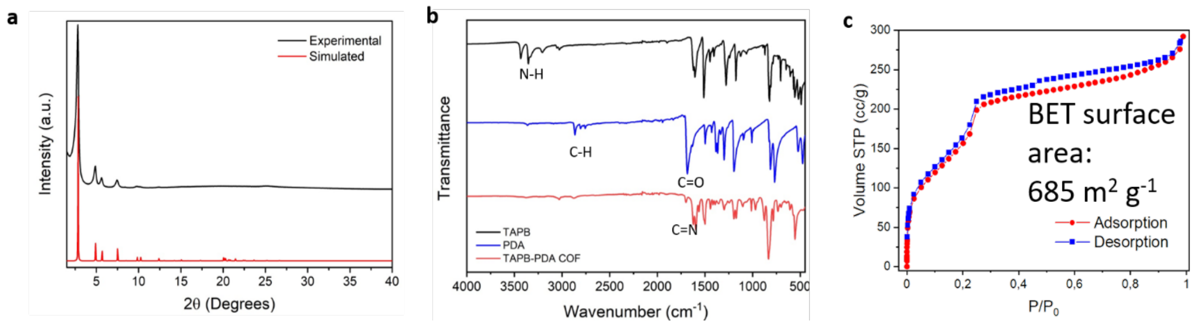

Figure S1. TABP-PDA COF structural analysis. **a:** Powder XRD after washing. **b:** FTIR of the precursors and the COF. **c:** BET surface area measurement for overall surface area analysis.

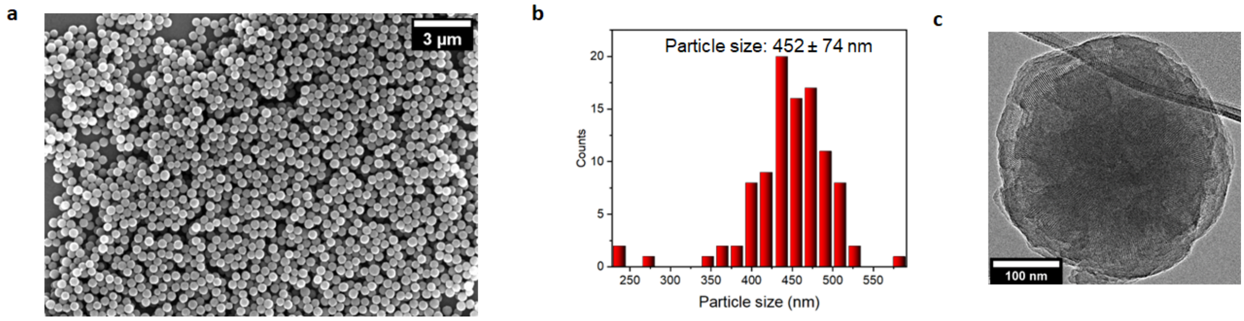

Figure S2. TABP-PDA COF particle morphology and structure. **a:** SEM image illustrating uniform size distribution of the washed COF microparticles. **b:** Particle size distribution showing high uniformity. **c:** TEM image showing a single COF nanoparticle consisting of crystalline domains with a lateral size of approximately 50 nm.

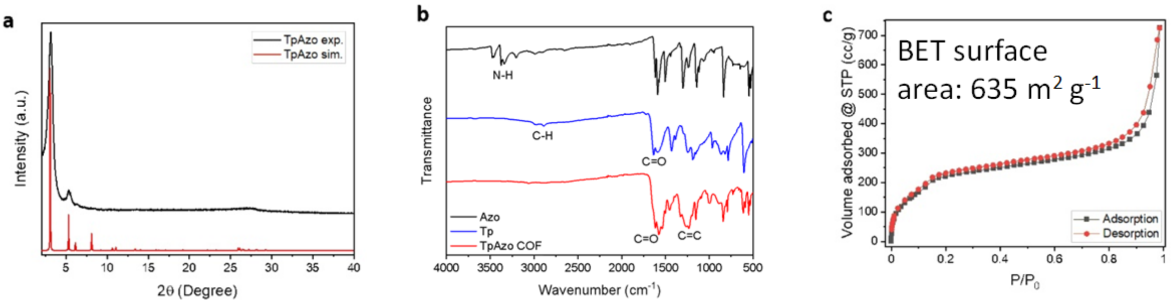

Figure S3: TpAzo-COF structural analysis. **a:** Powder XRD after washing. **b**: FTIR of the precursors and the COF. **c:** BET surface area measurement for overall surface area analysis.

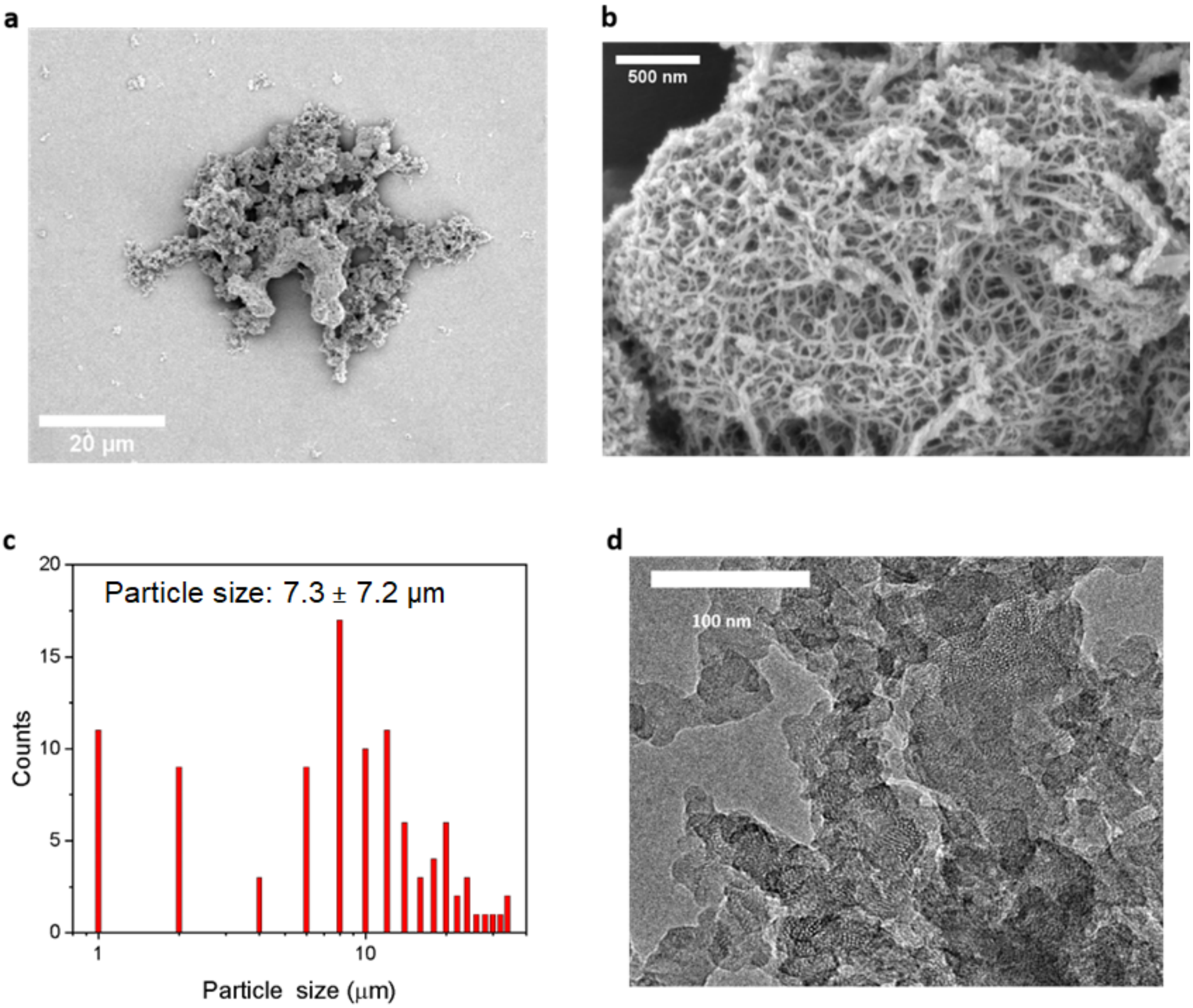

Figure S4. TpAzo-COF particle morphology and structure. **a:** SEM image illustrating the agglomerated structure of TpAzo-COF microparticles. **b:** SEM image (zoomed in) showing sponge-like inner structure with macropores. **c:** Particle size distribution showing non-uniformity of the particle agglomerates. The particle size ranges overall from 1 to almost 100 µm and is centered around 7.3 µm, with a standard deviation of 7.2 µm, as measured by SEM.. **d:** TEM image showing the interconnection of crystalline COF nanosheets with a domain size of approximately 50 nm or less.

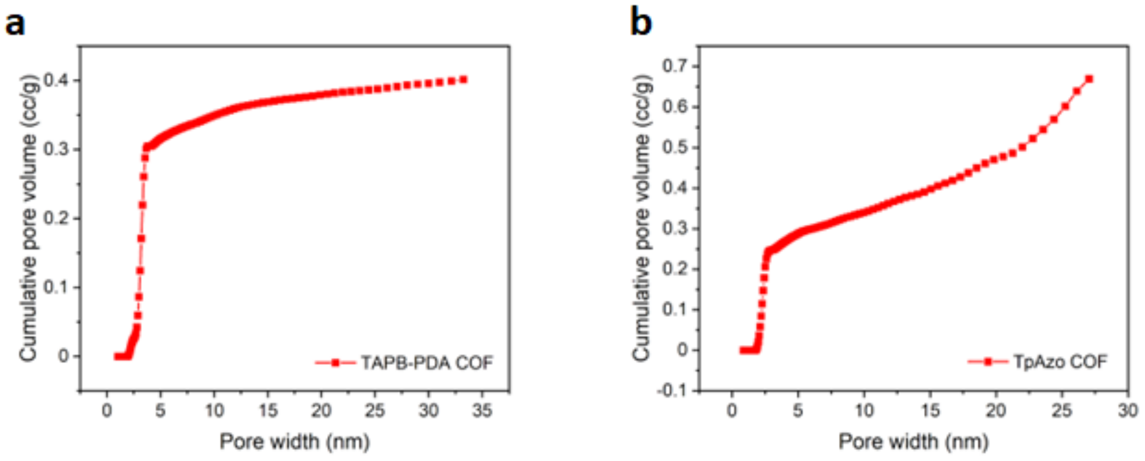

Figure S5. Cumulative pore volume results in the BET measurements for TAPB-PDA COFs (**a**) and TpAzo-COFs (**b**).

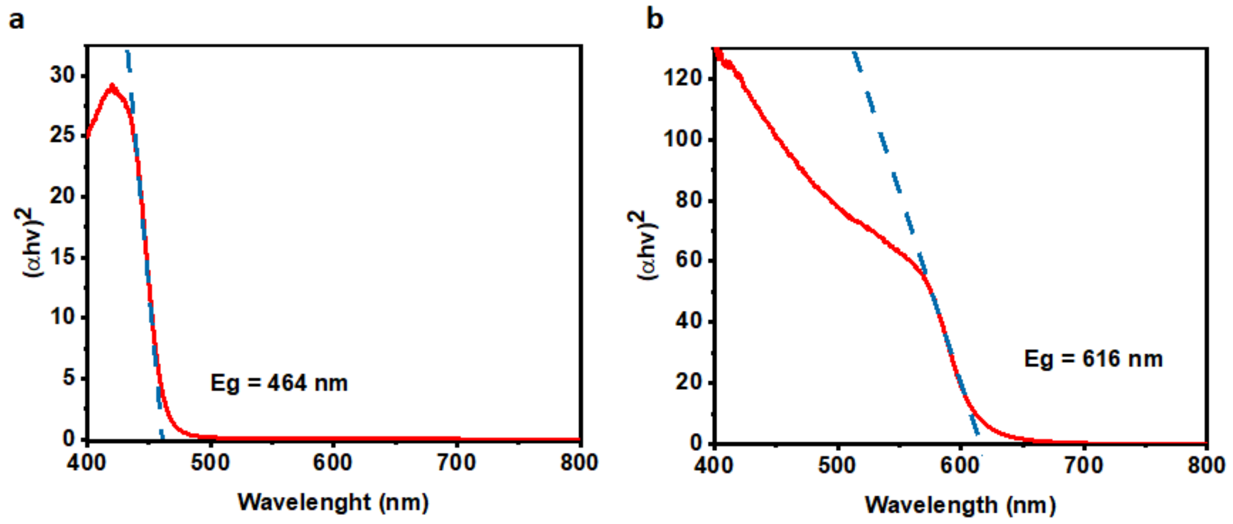

Figure S6. UV-Vis spectroscopy and Kubelka-Munck analysis of TAPB-PDA-COF **(a)** and TpAzo-COF **(b)**. They have an optical band gap of 2.70 eV and 2.01 eV, respectively, corresponding to 464 nm for TAPB-PDA-COF and 616 nm for TpAzo-COF.

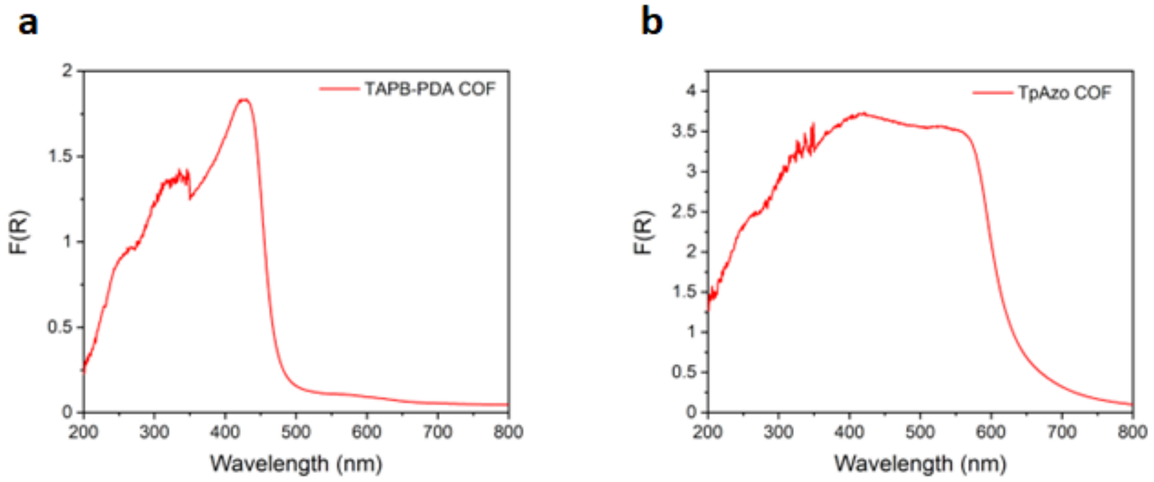

Figure S7. F(R) versus wavelength results for TAPB-PDA-COFs (**a**) and TpAzo-COFs (**b**).

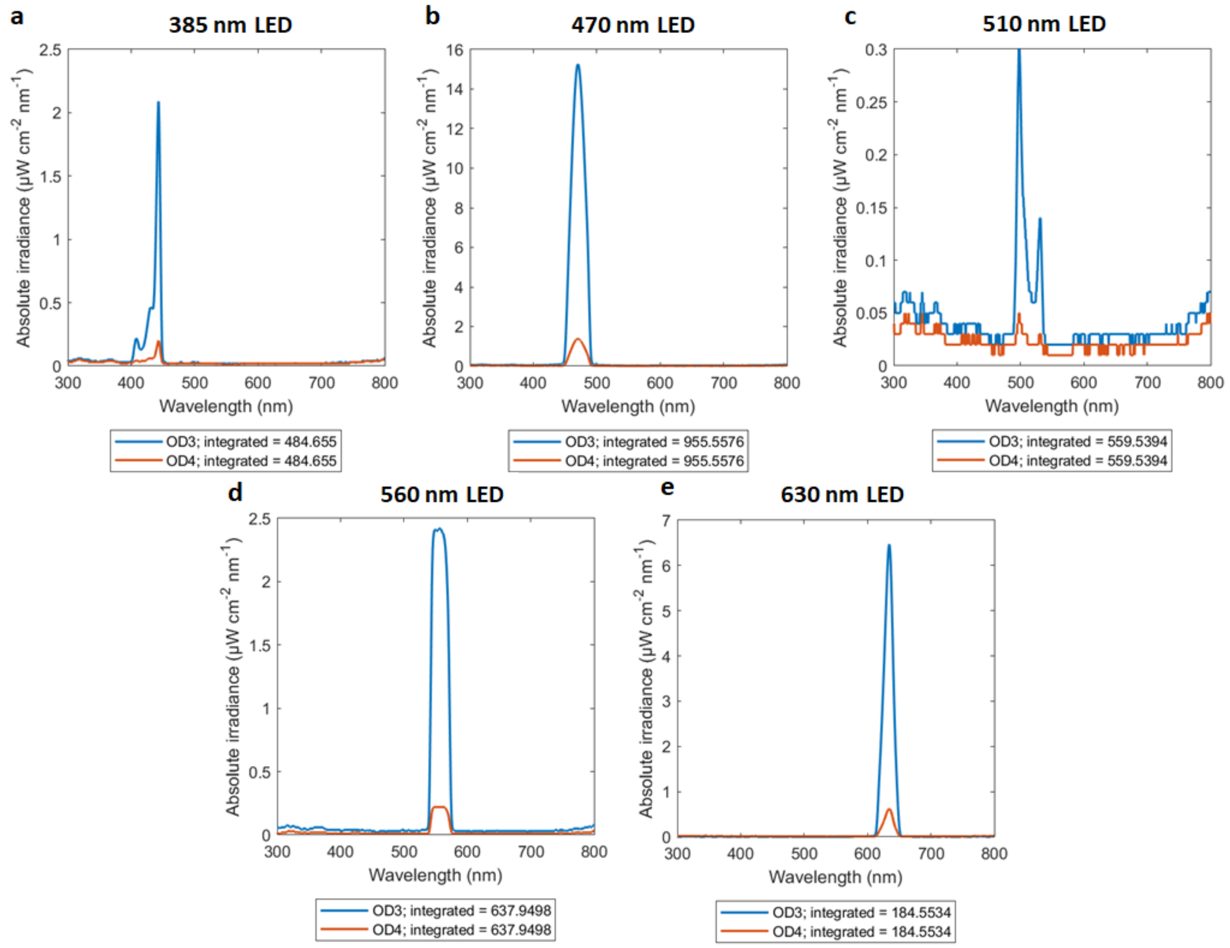

Figure S8. Wavelength spectra of the light-emitting diodes are used for the light-propulsion experiments, 385 (**a**), 470 (**b**), 510 (**c**), 560 (**d**), and 630(**e**) nm light sources. The reference spectra of the LED light sources, in the microscope, were measured with a calibrated extended-range spectrometer, using an integration time of 200 ms, a boxcar width of 5, and averaging over 5 scans. Two measurements were performed with different reflective neutral density filters (QD3 and QD4) with an optical density of 3 and 4 to check for consistency. The spot size (3 ± 0.5 mm diameter) of the light beam for the microscope is focused on the surface of the collimator optical fiber of the spectrometer, and the focus is validated with visualization of the field of view on the optical paper. See Table S1 for intensity details.

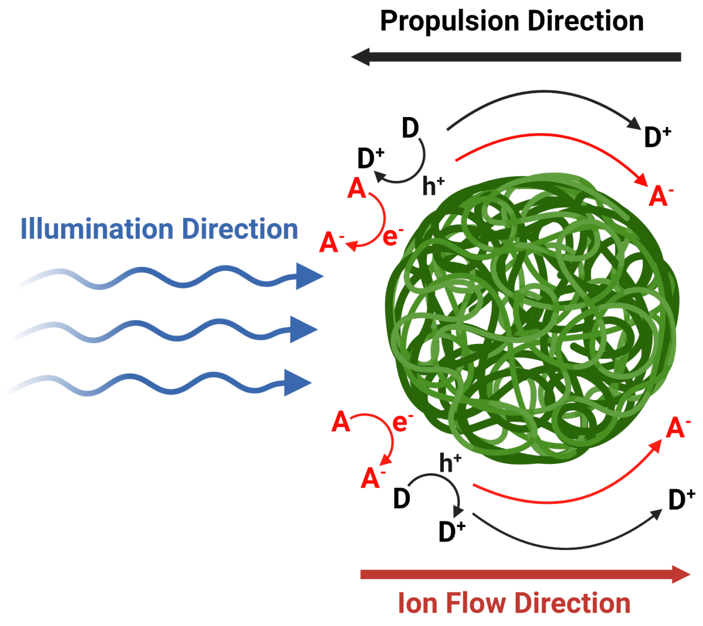

Figure S9. Proposed phototaxis mechanism for the COF-based microswimmers. Nanopores are not to the real scale. Density and particle shape are not perfectly spherical in reality. Phototaxis is caused by asymmetric illumination and photocatalysis, which results in ion flow around the particle. The movement of electron donors (D) and acceptors (A) (being oxidized and reduced, respectively) around the particle creates an ionic gradient between the illuminated and dark sides of the particle. h^+ and e^- represent the holes and electrons, respectively.

Central wavelength (nm)	Integral peak light intensity (mW/cm^2) with OD3 filter used for measurement	OD4 Integral peak light intensity (mW/cm^2) with OD4 filter used for measurement
385	24.7	32.6
470	387.8	350.3
510	6.1	14.3
560	69.1	64.3
630	117.0	114.0

Table S1. The peak light intensity measurement of the LED light sources in the microscope is used for the light-driven propulsion experiments. All peak light intensity measurements for the microscope were measured on the spot size (3 ± 0.5 mm diameter) of the light beam with a calibrated extended range spectrometer (OFX01341, Ocean Optics, FL, USA), equipped with a cosine corrector (3.9 mm diameter, 0.1195 cm^2 collection area) and an(integration time of 200 ms, a boxcar width of 5, and averaging over 5 scans.

Supplementary Videos

Video S1. Light-driven propulsion of 10 µg/ml TABP-PDA and TpAzo COF microswimmers inside distilled water with a 470-nm wavelength light source

Video S2. Phototaxis behavior of 10 µg/ml TABP-PDA and TpAzo COF microswimmers inside MEM using a directional 470-nm wavelength light source

Video S3. TABP-PDA COF (10 µg/ml) and TpAzo COF (10 µg/ml) microswimmer propulsion by light inside the porcine aqueous and porcine vitreous humor fluid

Video S4. Example 2D particle tracking analysis videos for TABP-PDA COF (10 µg/ml) and TpAzo COF (10 µg/ml) microswimmers in distilled water and aqueous humor

Video S5. Optical coherence tomography (OCT) imaging and guided trapping of TABP-PDA and TpAzo COF light-driven microswimmers inside the aqueous humor fluid

Video S6. Optical coherence tomography (OCT) imaging and guided propulsion of TABP-PDA and TpAzo COF light-driven microswimmers inside the anterior chambers of the porcine eye (ex vivo)
